# Keeping It Local: Dispersal Limitations of Coral Larvae to the High Latitude Coral Reefs of the Houtman Abrolhos Islands

**DOI:** 10.1371/journal.pone.0147628

**Published:** 2016-01-26

**Authors:** Kathryn L. Markey, Dave A. Abdo, Scott N. Evans, Cyprien Bosserelle

**Affiliations:** 1 Marine Ecology and Monitoring Section, Biodiversity and Biosecurity Branch, Department of Fisheries, Government of Western Australia, PO Box 20, North Beach, WA 6920, Australia; 2 Geoscience Division, Secretariat of the Pacific Community, Private Mail Bag, GPO, Suva, Fiji Islands; Department of Agriculture and Water Resources, AUSTRALIA

## Abstract

In 2011 the first recorded bleaching event for the high latitude Houtman Abrolhos Islands (HAI) coral communities was documented. This bleaching event highlighted the question of whether a supply of ‘heat tolerant’ coral recruits from the tropical north would be sufficient to provide a level of resistance for these reefs to future warming events. Using Lagrangian modelling we showed that due to its regional isolation, large-scale larval input from potential tropical northern source populations to the HAI is unlikely, despite the southward flowing Leeuwin current. Successful recruitment to artificial substrates was recorded following the bleaching event. However, this was negligible (0.4 ± 0.1 recruits per tile) compared to 2013 post impact recruitment (128.8 ± 15.8 recruits per tile). Our data therefore provides preliminary evidence suggesting that the connectivity of the HAI with coral communities in the north is limited, and population maintenance and recovery is likely driven primarily by self-recruitment. Given the low thermal tolerance of the HAI coral communities, the dominance of *Acropora*, and the apparent reliance on self-recruitment, an increased frequency of thermally anomalous conditions at the HAI (such as experienced in 2011) has the potential to reduce the long-term stability of the HAI coral populations and species that depend upon them.

## Introduction

Coral reef communities have shown incredible persistence in taxonomic composition and diversity during multiple episodes of global climate change [1]. Despite this long-term resilience, there has been an unprecedented decline and change in the comparative constancy in coral community composition in the past few decades [2]. This has been attributed to multiple causes including warming sea temperatures. Increasing sea temperatures have been directly linked to the occurrence of coral bleaching, and thermal bleaching events have resulted in significant mortality and dramatic shifts in coral community structure [3]. However, the majority of high latitude coral reefs (>26° latitude), such as those at the Houtman Abrolhos Islands (HAI), have been less affected by thermal bleaching compared to their tropical counterparts [4–6], leading to suggestions that they may be important refugia for tropical species in periods of warming [7, 8].

In Western Australia (WA), coral reef taxa (over geological time scales) have responded to a warming climate [7], with reefs at the edge of their distributions (e.g. high latitude reefs) suggested to have adapted to climate-induced increases in temperature [9]. On a more contemporary scale, a significant shift in the WA marine climate has been documented, with warmer water reaching further south [10], which has seen tropicalisation of the sub tropics and temperate reefs through changes to the range limits of species [11]. The capacity of the HAI to act as a coral refuge will depend on the stability of its coral population under future changes to the WA marine climate and its connectivity to the northern reef systems. An extremely strong La Niña event during the 2010/11 summer resulted in a record-strength poleward movement of warm tropical waters by the Leeuwin Current [12] and significant coral bleaching was recorded for the first time at the HAI in 2011 (~50% coral mortality across nine sites) [13]. The supply of recruits from the tropical north, which originate from warmer temperatures than found at the HAI (hereafter referred to as ‘heat tolerant’), may serve to improve the resistance of the HAI coral communities to increased temperatures. However, the increasing trend of warmer water at the HAI may further increase the possibility of thermal anomalies and subsequent bleaching [13]. The question is therefore raised as to whether a supply of ‘heat tolerant’ recruits from the tropical north will be sufficient to overcome the warming marine climate at the HAI, and hence provide an adaptive capacity to a changing climate.

At present, the extent of physical and ecological connectivity among WA reefs is unknown, but as the majority of coral larvae generally settle within one week of spawning [14, 15], Underwood et al. [16] hypothesised that coral systems in north-west Australia will be primarily replenished each generation by recruits that are produced locally, with coral dispersal distances ranging from ~10 km to ~50 km. This has also been observed along the eastern seaboard (e.g. between the Great Barrier Reef and higher latitude reefs like Lord Howe Island) where evidence of local recruitment has been observed [17, 18]. Given the isolated nature of the HAI, being ~240 km south from its nearest reported coral reef community neighbour (i.e. Shark Bay) [19], and a maximum reported larval competency period of up to 110 days (e.g. *Acropora valida*, see Table 1), it is predicted that localised production of coral recruits will be extremely important for replenishment of HAI coral communities. To test this, we used standard Lagrangian modelling to examine the likelihood of larval input to the HAI from external sources. The model was run over two years to represent normal Leeuwin Current conditions (Austral summer 2009/2010) and strengthened Leeuwin Current conditions (Austral summer 2010/2011). In addition, we recorded coral settlement rates on artificial substrates in 2011–2013 to quantify the number of recruits settling at the HAI [20]. The objective of the study was to use a combination of modelled dispersal patterns and empirical recruitment data to understand connectivity between the HAI with its northern counterparts, in a preliminary effort to estimate the potential resistance of the HAI coral communities to any future warming events.

**Table 1:** Review of larval competency of hard and soft corals.

Species	50% Mortality (DAS)	Max Longevity (DAS)	Reproductive Mode	Maximum competency period (DAS)	Reference
*Acanthastrea lordhowensis*	NR	78	Broadcast	78	Wilson and Harrison 1998 [62]
Acropora digitifera*	10	54	Broadcast	54	Nishikawa and Sakai 2005 [63]
Acropora formosa*	NR	23	Broadcast	NR	Harrison et al. 1984 [64]
*Acropora gemmifera*	14	60	Broadcast	<34	Baird 2001 [65]
Acropora hyacinthus*	NR	91	Broadcast	NR	Harrison et al. 1984[64]
Acropora hyacinthus*	21	38	Broadcast	NR	Nozawa and Okubo 2011 [66]
*Acropora japonica*	14	44	Broadcast	NR	Nozawa and Okubo 2011 [66]
Acropora latistella*	4	209	Broadcast	NR	Graham et al. 2008[32]
Acropora millepora*	14	110	Broadcast	<60	Baird 2001 [65]
*Acropora muricata*	16	50	Broadcast	NR	Nozawa and Harrison 2008 [67]
Acropora solitaryensis*	14,21	53	Broadcast	NR	Nozawa and Okubo 2011 [66]
Acropora tenuis*	25	69	Broadcast	69	Nishikawa et al. 2003[68]
Acropora valida*	16	130	Broadcast	<110	Baird 2001 [65]
Acropora valida*	14	50	Broadcast	NR	Nozawa and Harrison 2008 [67]
Cyphastrea serialia*	NR	26	Broadcast	26	Wilson and Harrison 1998 [62]
*Dendronephthya hemprichi*	NR	81	Broadcast	74	Ben-David-Zaslow and Benayahu 1998 [69]
*Dendronepthya hemprichi*	70	100	Brooding	65	Dahan and Benayahu 1998 [70]
Favia pallida*	19	195	Broadcast	NR	Graham et al. 2008[32]
Favites chinensis*	32	63	Broadcast	63	Nozawa and Harrison 2008 [67]
Goniastrea aspera*	138	215	Broadcast	NR	Graham et al. 2008[32]
Goniastrea aspera*	14	70	Broadcast	70	Nozawa and Harrison 2008 [67]
Goniastrea australiensis*	NR	56	Broadcast	<56	Wilson and Harrison 1998 [62]
Goniastrea favulus*	NR	60	Broadcast	NR	Babcock 1984 [71]
Goniastrea pectinata*	35	60	Broadcast	NR	Nozawa and Okubo 2011 [66]
*Goniastrea retiformis*	11	60	Broadcast	<36	Baird 2001 [65]
*Heliopora coerulea*	70	72	Brooding	30	Harii et al. 2002 [72]
*Heteroxenia fuscescens*	24–37	50	Brooding	49	Ben-David-Zaslow and Benayahu 1996 [73]
*Litophyton arboreum*	40	92	Brooding	<57	Ben-David-Zaslow and Benayahu 1998 [69]
Montastrea magnistellata*	124	244	Broadcast	NR	Graham et al. 2008[32]
*Nephthea sp*.	NR	57	Brooding	57	Ben-David-Zaslow and Benayahu 1998 [69]
Pectinia paeonia*	53	209	Broadcast	NR	Graham et al. 2008[32]
Platygyra daedalea*	3	60	Broadcast	<34	Baird 2001 [65]
Platygyra daedalea*	NR	124	Broadcast	105	Nozawa and Harrison 2000 [74]
Platygyra sinensis*	NR	15	Broadcast	15	Tay et al. 2011 [75]
Pocillopora damicornis*	100	100	Brooding	100	Harii et al. 2002 [72]
Pocillopora damicornis*		103	Brooding	103	Richmond 1987 [76]
Stylophora pistillata*	10	51	Brooding	51	[68]
*Xenia umbelata*	72	155	Brooding	76	Ben-David-Zaslow and Benayahu 1998 [69]

DAS = days after spawning; NR = not reported.

Asterisk (*) indicates species reported to occur at Houtman Abrolhos Islands [61].

## Materials and Methods

### Study location

**Fig 1:**
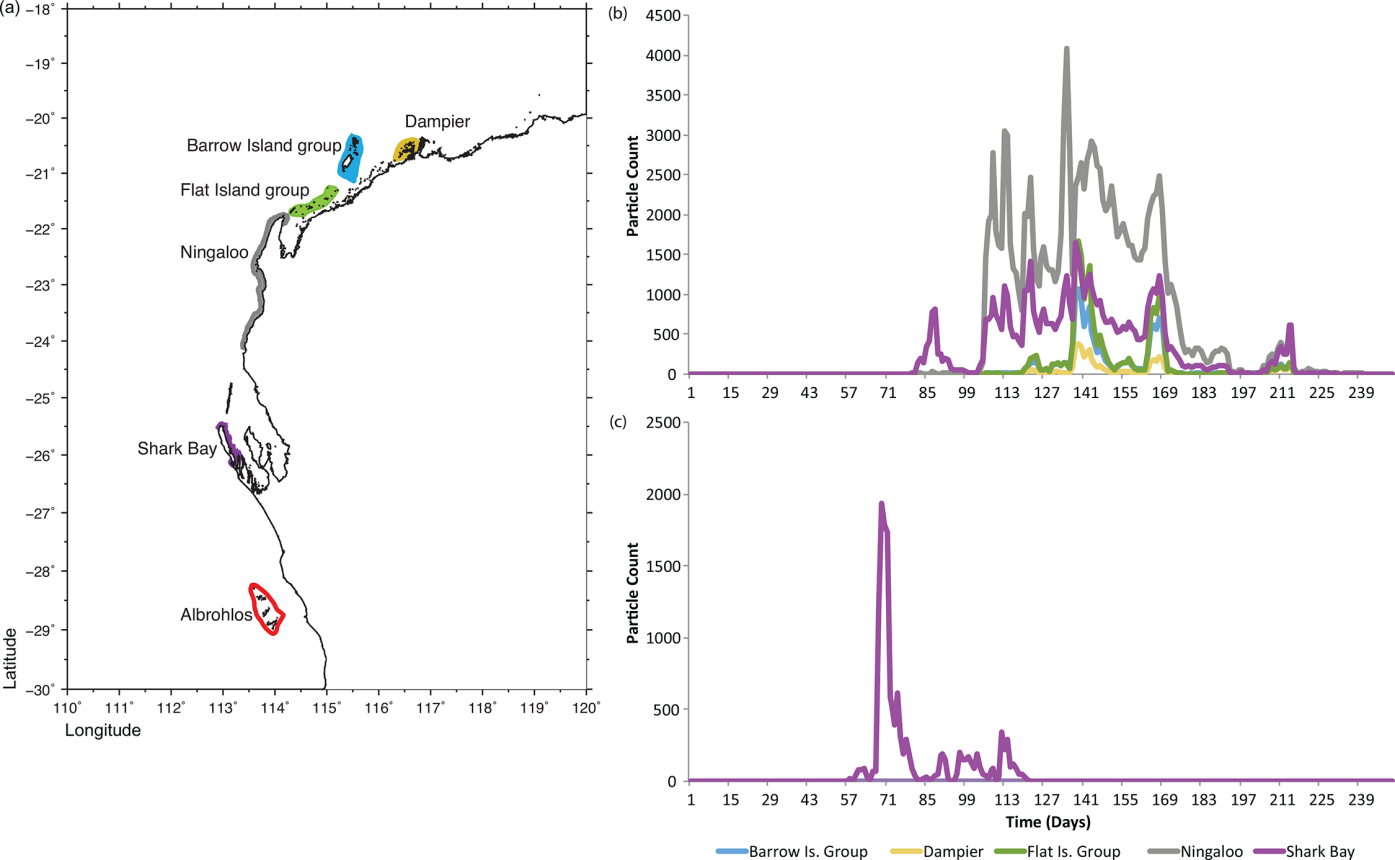
a) Location of potential northern seeding reefs and the HAI used in the dispersal modelling b) Simulated larval arrivals from the potential northern seeding reefs to the HAI in 2010 and in c) 2011.

The HAI are located on the edge of the WA continental shelf between 28°16’S and 29°00’S, in the pathway of the warm poleward flowing Leeuwin Current (Fig 1). Due to the influence of the Leeuwin Current transporting tropical marine fauna southwards, the HAI has an exceptional range of marine diversity (including 184 coral species from 42 genera), with tropical species co-existing with temperate species including habitat structuring alga [21].

### Particle dispersal model

Particle dispersal modelling was undertaken for 2010 (considered a ‘normal’ year) and 2011 (considered an ‘anomalous’ year). In this model, coral larvae were assumed to be passive neutrally buoyant particles. The dispersal model used a standard Lagrangian formulation [22, 23] of displacement that can be written as:

Δx=Up*Δt+K Eq (1)

where *x* is the particle position along a given axis (Latitude and longitude in our case) and Δ*x* is the particle displacement during a time step Δ*t*. Here the time step was chosen as 600 seconds. Up is the surface current speed at the location of the particle and *K* is the diffusivity that takes account of the random displacement of the particle due to turbulent eddies at a scale smaller than the hydrodynamics model resolution.

The eddy diffusivity can be calculated using the eq. proposed by Viikmae et al. [24]

$$\text{K}=\sqrt{\text{-}4{\text{E}}_{\text{h}}}\;\Delta \;\text{t}\;\log\left(1-R_{NA}\right)\;\cos\left(2\pi R_{NB}\right)$$ Eq (2)

where *E*_*h*_is an horizontal turbulent diffusion coefficient. Δ*t*is the model time step (here 3600 seconds) *R*_*NA*_ and *R*_*NB*_ are normally distributed random numbers. The horizontal turbulent diffusion coefficient is unknown but is assumed to be 1m^2^s^-1^. The surface current speed (Up) is calculated by interpolating the velocity from the hydrodynamics model both in space and in time. The interpolation is first on the particle position using a bi-linear interpolation of the gridded surface currents. Only the surface currents are taken into account in the interpolation and vertical movement along with settling and resuspension of larvae is not considered here. The particle age is kept and increases with the model progression. To allow a delayed released, particles are given a negative age and are only allowed to move according to Eq (1) when their age becomes positive.

This particle model was driven by current velocity output from hydrodynamic numerical models WASA for WA (for 2010) and OzROMS for Australia (for 2011) with a daily output on a curvilinear grid with resolution between 2–4 km [25, 26]. The hydrodynamic model simulates the full 3 dimension of the water column. However, only the surface layer was used to drive the dispersal model.

To determine potential seeding locations, the model was run backwards and areas of significant coral cover within the path of the released backwards-flowing particles within 250 days were considered potential seeding locations. At each of the potential seeding locations (Dampier Archipelago, Barrow Island group, Flat Island group, Ningaloo Reef, Shark Bay) 256,000 particles were seeded every day for 5 days following the 31st March (total 1,280,000 particles per location). The number of particles to reach the HAI area was then recorded over the following 250 days. While the exact coral spawning dates for 2010 and 2011 differed (30^th^ March 2010 and 18^th^ March 2011, respectively), for model consistency, the particle release time was kept constant to allow for exact comparison of the output.

### Recruitment tiles

Corals in WA are known to have a major spawning event in Austral autumn (March—April) [27], which coincides with a strengthening of the Leeuwin Current. As such, standard terracota recruitment tiles (12 x 12 x 1 cm) were deployed in January of 2011, 2012, and 2013 at 3 sites at the HAI following standard methodologies [28]. At each site, three replicate sets of tiles (5 tiles per replicate) were deployed just off the sea floor at between 8–10 m depth. Each year, the tiles deployed in January were retrieved in May, bleached, and transported to the laboratory. Newly settled corals were analysed using a binocular microscope examining the top and bottom surfaces of the tiles, and identified as Acroporidae, Pocilloporidae, Poritidae and other [29]. The recruitment data were Log (x+1) transformed prior to analysis, and a resemblance matrix of similarities was calculated using Euclidean distance. To examine changes in the number of recruits, a Permutational Analysis of Variance (PERMANOVA) [30] was performed with Year as a fixed factor, followed by pair-wise post-hoc tests. Sea surface temperature data was obtained from 4 January 2010 to 30 December 2013 from the National Oceanic and Atmospheric (NOAA) HAI virtual station at 28.5°S 114.0°E [31].

## Results and Discussion

Under normal Leeuwin Current conditions (as per 2010 in our model), the first of the simulated propagules arrived at the HAI from its nearest neighbour Shark Bay after 57 days (Fig 1B). However, this only represented <0.01% of the propagules received from Shark Bay. The next phase of arrivals from Shark Bay occurred at 76 days, and coincided with the first arrivals from Ningaloo at 77 days (Fig 1B). Fifty percent of arrivals had occurred after 138 days and 141 days after release from Shark Bay and Ningaloo, respectively, although the rate of arrivals was 125% greater from Ningaloo compared to Shark Bay (Fig 1B). The first of the simulated propagules arrived at the HAI from the Barrow Island group after 81 days (Fig 1B), but again only represented <0.01% of the propagules received from the Barrow Island group. The next phase of arrivals from the Barrow Island group occurred at 104 days, and coincided with the first arrivals from the Dampier Archipelago and the Flat Island Group at 105 days (Fig 1B). The simulated propagules from the Flat Island group, the Barrow Island group, and the Dampier Archipelago were bimodal, peaking at ~140 days and ~160 days from release (Fig 1B).

During 2011, the Leeuwin Current was at a record strength associated with a strong La Niña event during the 2010/11 summer [12]. Under these conditions, the simulated propagules starting arriving at the HAI from Shark Bay at 56 days, with the peak arrival between 65–80 days (Fig 1C). There were no simulated propagule arrivals from any of the other four potential source locations under the strengthened Leeuwin Current conditions for the full 250 days of the model (Fig 1C).

While some coral species have larvae that are reported to survive for up to 200 days [32] and complete metamorphosis up to 100 days after spawning under laboratory conditions [33], the simulated propagule arrival times of between 80–180 days in our models is beyond the upper competency periods of most coral species [32, 34]. Moreover, the basic Lagrangian simulations used did not allow for particle dilution or mortality, which has been shown to reduce larval (simulated particle) movement by up to 9-fold [35]. Entrapment and early settlement on natal or nearby reefs [16, 36], mortality, predation and dilution prevent much long distance larval transport [37], and the ecological likelihood of external recruitment from sources beyond 50 km is expected to be limited [38]. Studies using genetic, oceanographic, or modelling techniques to infer coral dispersal distances have found that larvae routinely disperse ≤10s of kms, with dispersal >50–100s of kms generally restricted to occasional genetic connections (see summary in Jones et al. 2009). Given that our models represent an overestimation of the connectivity between the HAI and its northern neighbours >240km away, the capacity of the northern source locations to supply ‘heat tolerant’ recruits to the HAI, or sufficient larvae to facilitate recovery following significant mortality of the local population is likely to be limited.

**Fig 2:**
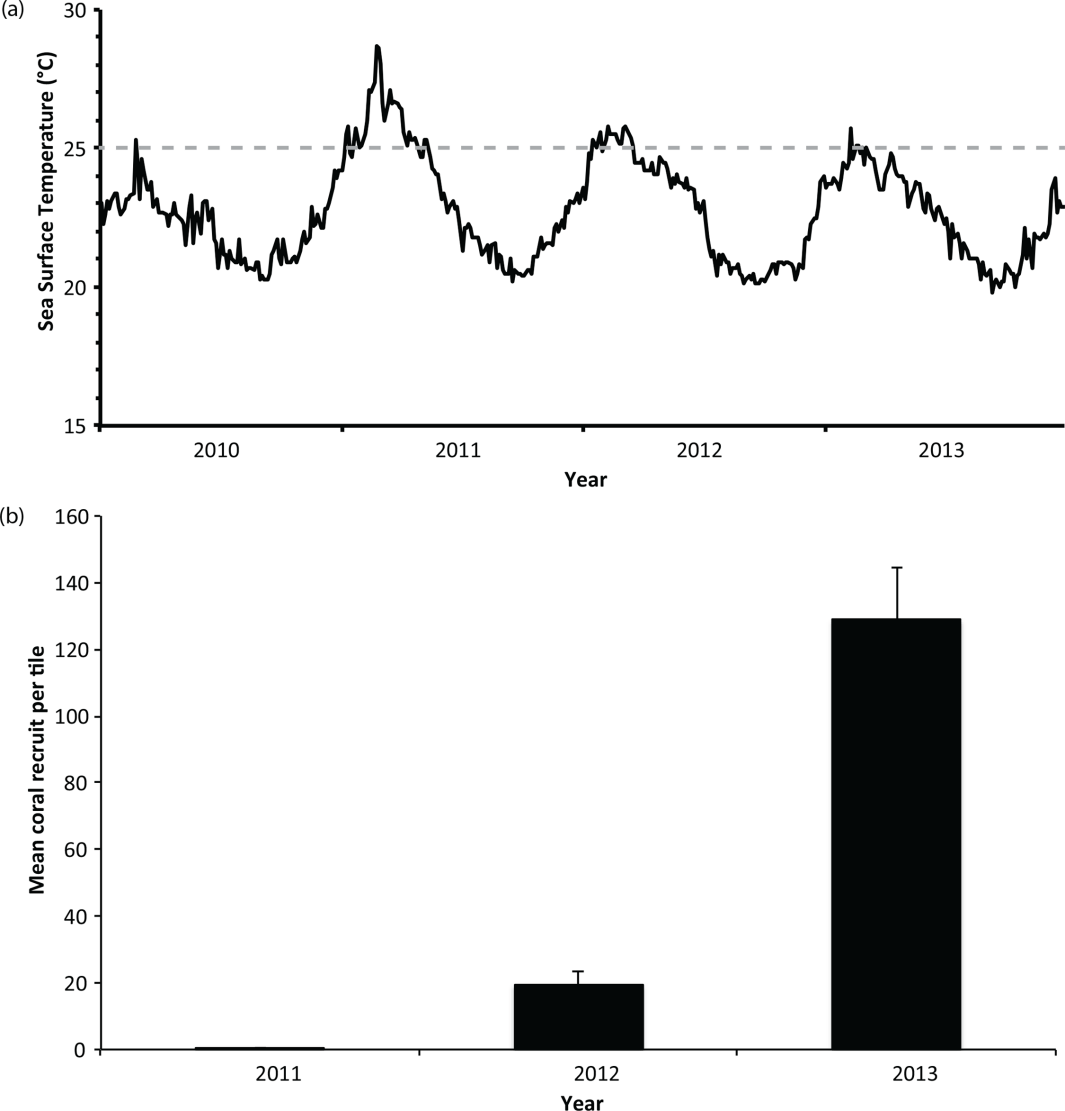
a) NOAA Sea surface temperature data from 2010–2013. Note 2010 represents a typical temperature profile for HAI, unlike the anomalous temperatures seen in 2011. b) Average number of coral recruits tile^-1^ (± Standard Error) at the HAI following the autumn mass spawning from 2011–2013.

The results of our model outputs therefore suggest that the population dynamics of the coral communities of the HAI are largely driven by self-recruitment rather than frequent input of larvae from external northern population sources. This reinforces a study on the population genetic structure of *Pocillopora damicornis* at the HAI that indicated that the populations were primarily self-seeding [39]. Further, a recent study by Thomas et al. (2015) found a complex localised genetic structure within *Acropora spicifera* populations at HAI, suggesting isolated local populations and enhanced self-recruitment within the island groups at HAI [40]. The Acroporidae largely dominate the coral community of the HAI [41] and this was reflected in the recruitment data, where >95% of the recruits that settled on the artificial tiles were Acroporidae. The recorded recruitment rates differed significantly among all years (Table 2). During the anomalous summer 2010/2011, where temperatures reached up to 5°C above long-term seasonal averages [12] (Fig 2B), there was almost complete recruitment failure on the artificial tiles in 2011 (0.4 ± 0.1 recruits per tile; Fig 2B). The number of recruits per tile increased in 2012 (19.5 ± 4.2 recruits per tile; Fig 2B), with the highest number of recruits per tile recorded in 2013 (128.8 ± 15.8 recruits per tile; Fig 2B).

**Table 2:** Permutational Analysis of Variance for coral recruits tile^-1^ at the HAI following the autumn mass spawning from 2011–2013.

**Main test**						
**Source**	**df**	**SS**	**MS**	**Pseudo-F**	**P(perm)**	**perms**
Year	2	379.02	174.51	69.622	0.0001	9963
Res	177	443.65	2.5065			
Total	179	792.67				
**Post-hoc test (Year)**						
	2011		2012		2013	
2011	X					
2012	0.0001		X			
2013	0.0001		0.0001		X	

The anomalous temperatures recorded in the summer 2010/2011 that resulted in bleaching and ~50% mortality of the adult coral population at the HAI [13] was likely to be a factor in the poor recruitment recorded in 2011. The loss of adult brood stock has been shown to disproportionately affect recruitment success on isolated reefs [42], and coral larval survival [43], recruitment, and post settlement survival [44] can be negatively affected by increased temperatures. Coral bleaching has also been shown to reduce coral fecundity in some species [45–47]. Thus, the lower recruitment rates in 2012 may also be related to reduced reproductive output following the bleaching in the previous year, and/or due to temperature-related effects on the larvae as the sea surface temperatures in 2012 were also above seasonal averages prior to and including the spawning period (Fig 2A).

In contrast to the low rates of recruitment in 2011 and 2012, the 2013 recruitment rate (equivalent to 3832 recruits m^-2^ year^-1^) was higher than that reported for sub-tropical reefs [48] and more akin to rates recorded on the well connected central Great Barrier Reef [49–51]. Interestingly, during the 2013 spawning period, the Leeuwin current was at a relatively low strength associated with a neutral ENSO, whereas in 2011 (strong La Niña) and 2012 (moderate La Niña) the Leeuwin Current flowed strongly past the HAI. A strong southerly current through the HAI during spawning time (as experienced in 2011 and 2012) could potentially lessen the residence time of locally produced larvae, which may serve to reduce self-recruitment if the larvae are swept off their natal reefs before settlement competency is reached [52]. Such an effect may help in partly explain the low recruitment seen in 2011 and 2012. However, increased water temperatures have recently been shown to speed up competency in some coral species [53] which could help to counter the effect of decreased residence times. Strong La Niña events and associated strong Leeuwin currents therefore appear to have the potential to result in both thermal stress to the adult coral community and a reduction of larval retention at the HAI. Additional years of recruitment analysis and modelling of larval residence times at the HAI would be necessary to fully elucidate this pattern.

The recovery of the HAI coral population following the 2011 bleaching may therefore be longer than would have initially been expected due to the limited connectivity with the northern reef systems and localised genetic patchiness [40], the potential reduction of recruitment rates due to thermal stress on the adults, and/or the variable oceanographic conditions. This is of particular importance for populations in the Pelsaert Group, which appear to be isolated from the other island groups at the HAI [40]. The stability of the HAI coral population is therefore likely to depend on the frequency of future disturbances, as well as the survival and growth of the adult population following disturbance and sufficient successful recruitment. The HAI coral communities are largely comprised of branching *Acropora*, an important component of the structural complexity and reef building capacity of the HAI [54, 55]. Adult *Acropora* are extremely fecund [56] and grow rapidly [57]. However, they are also known to be particularly thermally sensitive [58, 59], and the HAI coral communities have recently been shown to have a low bleaching threshold [13]. With the documented trend of increasing marine temperatures it is likely that future bleaching will be recorded at the HAI, and as recruitment rates are reduced when the proportion of the population fecund falls below 80% [49], significant bleaching-associated mortality in the future has the potential to reduce local larval supply at the HAI. Moreover, given the location of the HAI in a tropical/temperate mixture zone, competition with coexisting macroalgal communities following disturbance could decrease suitable settlement substrate and post settlement survival [8, 60].

The results presented here suggest that the connectivity of the HAI to coral communities to the north is limited, and that self-recruitment among the island groups of HAI is is likely the primary source of coral recruits. The limited input of ‘heat tolerant’ recruits into the HAI coral population is unlikely to provide an increased resistance to future thermal stress, and if temperature pulses akin to those experienced in the summer 2010/2011 increase in frequency, future coral bleaching events are likely. Future bleaching events have the potential to result in reduced coral cover and abundance and a decreased supply of new recruits from natal sources. Given the dominance of *Acropora* and the apparent reliance on self-recruitment of the HAI coral communities, an increased frequency of thermally anomalous conditions at the HAI has the potential to reduce the long term stability (i.e. community structure, hard coral cover) of the HAI coral populations.
